# Emergent Presentation of Decompensated Mitral Valve Prolapse and Atrial Septal Defect

**DOI:** 10.5811/westjem.2015.2.25469

**Published:** 2015-04-06

**Authors:** Jessie Kang, Bijon Das

**Affiliations:** Dalhousie University, Department of Emergency Medicine, Halifax, Nova Scotia

## Abstract

Mitral valve prolapse is not commonly on the list of differential diagnosis when a patient presents in the emergency department (ED) in severe distress, presenting with non-specific features such as abdominal pain, tachycardia and dyspnea. A healthy 55-year-old man without significant past medical history arrived in the ED with a unique presentation of a primary mitral valve prolapse with an atrial septal defect uncommon in cardiology literature. Early recognition of mitral valve prolapse in high-risk patients for severe mitral regurgitation or patients with underlying cardiovascular abnormalities such as an atrial septal defect is crucial to prevent morbid outcomes such as sudden cardiac death.

## CASE REPORT

A 55-year-old man presented to the emergency department (ED) with abdominal discomfort and shortness of breath in obvious visible distress. He had a two-week history of abdominal discomfort. He had no other significant past medical history. He was seen at a walk-in clinic earlier on the same day due to chest discomfort, and was unable to speak in full sentences. He was sent to the ED by the general practitioner due to increasing shortness of breath. His social history was significant for 3–4 glasses of wine a night on weekdays, and more on weekends. He did not have a family history of heart disease.

On examination, his heart rate was 180 beats/min, systolic blood pressure was 80mmHg, respiratory rate was 30 breaths/min, temperature was 36.4°C, and oxygen saturation was 78% on room air. Upon auscultation, his S1 and S2 were normal and S3 and S4 were absent. He had a systolic murmur of grade 3/6 at the apex. Initial electrocardiogram (EKG) showed the patient in rapid atrial fibrillation with a ventricular response rate of 168 beats per minute. Some nonspecific ST and T wave abnormalities were found. Chest radiography showed markedly increased cardiothoracic ratio and clear lung fields. On laboratory workup, he was jaundiced with total bilirubin of 42umol/L, and a troponin level of 101ng/L. He was seen by cardiology and given an initial diagnosis of cardiomyopathy potentially alcohol induced. It was difficult to determine the primary reason for his presentation: possibilities of pulmonary embolism, ruptured chordae with mitral regurgitation (MR), and cardiomyopathy were considered. Plan of action was to electrically cardiovert if his condition deteriorated.

In the ED he was administered dopamine intravenously (IV) (5mcg/kg), which was subsequently increased to 7.5mcg/kg as his systolic blood pressure was difficult to maintain. He was also administered metoprolol 5mg IV and digoxin 0.5mg IV for rate control.

A transthoracic echocardiogram was performed, which revealed a normal left ventricular cavity size with estimated ejection fraction of 50–55%. The posterior mitral leaflet was determined to be flail with a ruptured mitral valve chordae, and there was severe MR.

The patient was admitted to the coronary care unit with an oxygen saturation in the 80s and extreme shortness of breath, diaphoresis and cyanosis of the extremities. Continuous positive airway pressure (CPAP) therapy was administered; however, he became increasingly anxious and dyspneic. A transesophageal echocardiogram revealed severe mitral regurgitation with P2 prolapse secondary to ruptured cords. Left ventricle function was poor at an ejection fraction of 30%, and the right ventricle was almost akinetic. He was subsequently intubated and evaluated in the catherization lab where upon selective coronary angiography, left and dominant right coronary arteries were determined to be normal. An intraaortic balloon pump was placed for surgery and he was transferred to the operating room on an emergent basis due to critical decompensation to undergo a mitral valve repair with a P2 resection and a 34mm annuloplasty ring.

Direct visualization of the mitral valve showed a posterior leaflet prolapse secondary to ruptured cords, which took up about one third of the posterior leaflet. There was an atrial septal defect (ASD) noted with some right to left shunting. The right atrium was large and pressurized. The posterior leaflet was resected and a 34 mm annuloplasty ring was put in place. The atrial septal defect was closed through the left atrium.

The patient tolerated the surgery well and returned to cardiovascular intensive care unit in good condition. He was found to be in atrial fibrillation post-op, which was rate controlled with nadolol. He was also started on warfarin for his atrial fibrillation. Upon discharge, he was given amiodarone to be tapered off and discontinued in the subsequent couple of weeks. Follow up at the outpatient cardiovascular clinic was unremarkable. He had no complaints or complications from the surgery and no further follow up was arranged.

Post-surgical pathology of the posterior leaflet revealed myxomatous mitral valve disease, with diffuse rubbery thickening.

## INTRODUCTION

Mitral valve prolapse (MVP) is a very common valvular abnormality that is likely to be an incidental finding on auscultation in the ED as it is often asymptomatic in patients.^1^ Patients with non-specific clinical features such as dyspnea, tachycardia and abdominal pain would have a wide differential diagnosis. It is crucial to identify individuals at increased risk for MVP or those with accompanying cardiac abnormalities such as an ASD to prevent serious complications such as severe MR and sudden cardiac death (SCD). Although secondary MVP has been associated in patients with ASD, our patient presented with a unique presentation of a primary MVP with an ASD in a severely decompensated state.^1^ This case report offers a different perspective of a patient in acute, severe distress with a primary MVP with an ASD infrequently reported in cardiology literature.

## DISCUSSION

MVP is a multifactorial valvular abnormality that can be caused by histological abnormalities or valvular tissue, geometric disparities between the left ventricle and mitral valve or various connective tissue disorders such as Marfan syndrome and Ehlers-Danlos syndrome.^1^ This diagnosis is typically detected by auscultation with systolic clicks or mid to late systolic murmur, as most patients with MVP are asymptomatic.^2^ As such, patients are unlikely to present to the ED with problems specific to MVP unless they develop serious complications such as severe MR or SCD.^1^

Myxomatous degeneration of the mitral valve is the most common pathophysiological basis for MVP, producing characteristic histologic changes also known as primary or classic MVP.^1^ However, secondary or non-classic MVP can occur in those with histologically normal valves, and is associated in 50–80% of patients with unrepaired secundum ASD.^1^ The underlying mechanism between classic and non-classic MVP differ: in the classic MVP, there is characteristic myxomatous degeneration of the valve with leaflet thickening and redundancy that appears to be due to a dysregulation of the components of the extracellular matrix.^1^ Comparatively, non-classic MVP can be attributed to imbalance of geometric features between the mitral valve and the left ventricle that govern the mechanical function of the mitral valve:, such as LV size, mitral annular dimensions, and the leaflet size.^3^

Regardless of the underlying mechanism, patients with MVP typically present with atypical chest pain, dyspnea, palpitations, syncope and anxiety, as well as lower blood pressure and non-specific T-wave abnormalities on EKG.^4^ Some of the clinical features of mitral regurgitation secondary to mitral valve prolapse include various clinical manifestations such as sudden onset dyspnea, fever, cough and chest pain.^5^ Often these presentations are non-specific and can be mistaken for other common emergency conditions such as pulmonary embolism, acute coronary syndrome or exacerbation of chronic obstructive pulmonary disease. Initial misdiagnosis of acute flail mitral valve causing severe mitral regurgitation is not infrequent and can result in morbid outcomes.^5^ It is therefore crucial to raise awareness of this clinical entity and to better identify mitral valve prolapse in those with atypical symptoms. This is especially true in patients who are high risk for severe MR such as presence of thickened leaflets, posterior leaflet prolapse and increased left ventricular dimensions, and those patients with other cardiovascular abnormalities such as ASD.^1^ Clinical course of MR and MVP are altered by the presence of ASD. Some patients with severe MR may not manifest typical symptoms of MR because the ASD may unload the left atrium making prompt diagnosis a challenge.^6^ Interestingly, in our patient, his long-standing ASD caused reverse shunting due to higher pressure in the right atrium than in the left atrium, resulting in partially deoxygenated blood pumping out of the ventricles. This, in combination with the flail mitral valve resulting in poor cardiac output, led in his severely decompensated state.

Management of this presentation in the ED setting involves stabilizing the patient in preparation for surgery. Intravenous vasodilators such as nitro may be given to reduce the MR by reducing the systemic vascular resistance and improving the mitral valve competence. This is, however, limited in a hypotensive patient with cardiogenic shock.^7^ Intraaortic balloon pump may be used as a temporary measure to reduce systemic resistance thus improving cardiac output, without a reduction in mean arterial pressure.^7^ Management involves use of oxygen or ventilatory support to improve hypoxemia. Cardiogenic shock requires fluid restriction although with poor right ventricular function, fluid administration may be required to increase preload.^8^ Treatment with inotropes will increase contractility of the heart and increase cardiac output, while decreasing afterload.^8^

In this case, we observed a patient with a myxomatous degeneration of the mitral valve with MR secondary to MVP. He also has an ASD, which is a risk factor of MVP independent of the myxomatous histological nature of the mitral valve. This led to his acute, distressed initial presentation that was not characteristic of that of a mitral valve prolapse. Early recognition of MVP in high-risk patients for severe MR or patients with underlying cardiovascular abnormalities is crucial to prevent morbid outcomes.
